# Enhancing Diagnostics in Orthopedic Infections

**DOI:** 10.1128/jcm.02196-21

**Published:** 2022-03-10

**Authors:** Eibhlin Higgins, Gina A. Suh, Aaron J. Tande

**Affiliations:** a Division of Infectious Diseases, Mayo Clinic, Rochester, Minnesota, USA; Vanderbilt University Medical Center

**Keywords:** septic arthritis, osteomyelitis, PJI, bone and joint infection, orthopedic infection, prosthetic joint infection

## Abstract

Accurate diagnosis of orthopedic infection is crucial in guiding both antimicrobial therapy and surgical management in order to optimize patient outcomes. A variety of microbiological and nonmicrobiological methods are used to establish the presence of a musculoskeletal infection. In this minireview, we examine traditional culture-based and newer molecular methodologies for pathogen detection, as well as systemic and localized assays to assess host response to maximize diagnostic yield.

## INTRODUCTION

**FIG 1:**
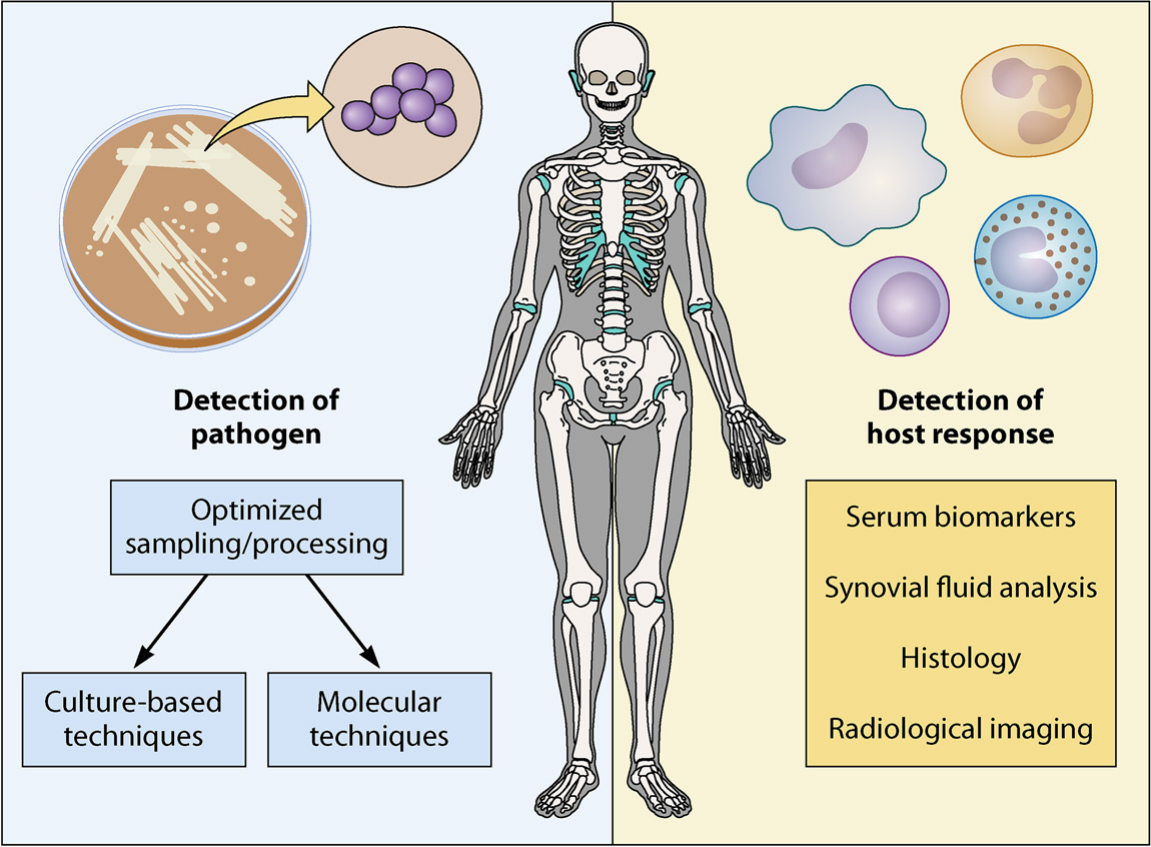
Principles of diagnostics in orthopedic infections.

The clinical spectrum of orthopedic infection is broad, and enhancing diagnostics is crucial to ensure accurate diagnosis, treatment, and improved outcomes. Prompt recognition of the clinical syndrome with appropriate diagnostic sampling and treatment with antimicrobial therapy are cornerstones of management. Obtaining representative samples can be challenging and often requires invasive sampling. Host factors such as comorbid inflammatory conditions and immunosuppressive medications make recognizing and detecting infection more difficult. The central tenets of diagnostics in orthopedic infection revolve around two core principles: detection of the pathogen and detection of the host inflammatory response (Fig. 1). In general, accurate diagnosis hinges upon a patient-centric combination of these approaches.

## PATHOGEN DETECTION

**Optimizing culture-based techniques.** Optimal sampling is crucial in the diagnosis of bone and joint infections. Peripheral blood cultures are an important diagnostic tool, but confirming a diagnosis of orthopedic infection generally requires synovial fluid, bone, or periprosthetic tissue sampling. The goal is to obtain samples in a way which minimizes contamination by skin flora. Samples should be taken using strict aseptic technique and avoid passage of the needle through sinus or fistula tracts, which may lead to contamination. In the case of prosthetic joint infections (PJI), multiple periprosthetic samples should be obtained using separate sterile instruments to avoid cross contamination. Larsen et al. (1) outlined an “all in a box” approach as a logistical tool to improve sampling process where the necessary surgical implements, transport vials, and labels necessary are organized in a kit to standardize sampling processes. Use of swabs for culture specimens is unhelpful in diagnosis of orthopedic infection, as the sensitivity is lower than that of tissue samples (2) and microbiologic concordance of superficial swabs with deeper samples is poor (3). Sensitivity of Gram stain for pathogen detection in orthopedic samples is low (4). Fungal and mycobacterial cultures should be done on orthopedic samples in select cases based on clinical suspicion and are not necessary as a routine practice (5). Presampling antibiotic therapy reduced yield of culture specimens and is the most important risk factor for culture-negative infection (6). Whenever possible, systemic antibiotics should be withheld for at least 2 weeks prior to culture ascertainment.

Distinguishing contaminants from true pathogens is particularly challenging with PJI where common commensal organisms can also be implicated in infection. Identification of these organisms in multiple separately obtained samples may help to distinguish pathogen from contaminant. In their prospective study to evaluate the optimum number of samples required to diagnose PJI, Atkins et al. (7) used histology of samples from patients undergoing revision surgery as a reference standard for infection diagnosis. Mathematical modeling determined that obtaining 5 to 6 surgical specimens for culture was necessary to obtain acceptable sensitivity and specificity. More recent studies (8, 9) utilized clinical rather than histopathological criteria as well as inoculation of tissue samples into blood culture bottles. While five tissue samples had the highest sensitivity in the study by Peel et al. (8), this was at the cost of specificity. Using Bayesian latent class modeling, they demonstrated the greatest accuracy for PJI diagnosis when three periprosthetic tissue specimens were obtained and inoculated into blood culture bottles. Bémer et al. (9) concluded that four samples instead of five had no impact on the clinical effectiveness of the microbiological diagnosis for PJI. Current Infectious Diseases Society of America (IDSA) guidelines for PJI recommend obtaining at least three and optimally five or six periprosthetic samples (10).

Inoculation of synovial fluid samples into blood culture bottles instead of agar has been demonstrated in multiple studies to increase yield (11, 12). Given that the microbial load in synovial fluid may be low, it is possible to inoculate larger amounts of fluid into blood culture bottles. Lytic agents contained in blood culture bottles may allow for detection of phagocytized bacteria, and the dilutional effect of placing the sample in a liquid medium may reduce inhibitory effects (12). Inoculation of periprosthetic tissue samples (13) into blood culture bottles has also been shown to increase yield.

Duration of bacterial culture incubation is of particular interest in PJI where slow-growing organisms such as Cutibacterium acnes may be implicated. A study of periprosthetic samples at revision arthroplasty in 2008 found that a substantial proportion of patients (26.4%) were classified as being infected when the period of culture was 14 days but would not have been classified as such had the duration of culture been only 7 days (14). A more recent retrospective study (15) found that an incubation period of 7 days was sufficient to identify 56 of 58 cases (96.6%). However, there was a small number of upper limb revision arthroplasties included in this cohort, and C. acnes was the causative agent in only 3 of the 58 cases. A study from Jeverica et al. (16) looked specifically at *C. acnes* isolates (*n* = 99) from orthopedic samples and compared anerobic blood culture bottle inoculation (using Bactec, Lytic/10 Anaerobic/F, Plus Anaerobic/F and BacT/Alert) with culture utilizing conventional media (thioglycolate broth, Schaedler agar, and chocolate agar). They found that anaerobic Schaedler agar and thioglycolate broth detect *C. acnes* faster and more reliably than automated blood culture systems. Thus, optimal culture methods for periprosthetic joint samples likely require a multimodal approach.

The role of biofilm in orthopedic implant-related infections can make obtaining representative samples challenging. Biofilms are intricate communities of microorganisms encased within an extracellular matrix which attach to foreign surfaces or, in relation to orthopedic infection, implants (17). Within biofilms, bacteria can evade the host immune response and are relatively protected from standard antibiotic therapy. Biofilm also complicates the diagnosis of infection, as the involved pathogens are concentrated within the biofilm on the surface of the prosthesis and can elude detection by culture of periprosthetic tissue or synovial fluid. Disrupting the bacteria within biofilm from the surface and thus enabling detection and enhancing sensitivity of diagnostic techniques is the premise underlying sonication.

**FIG 2:**
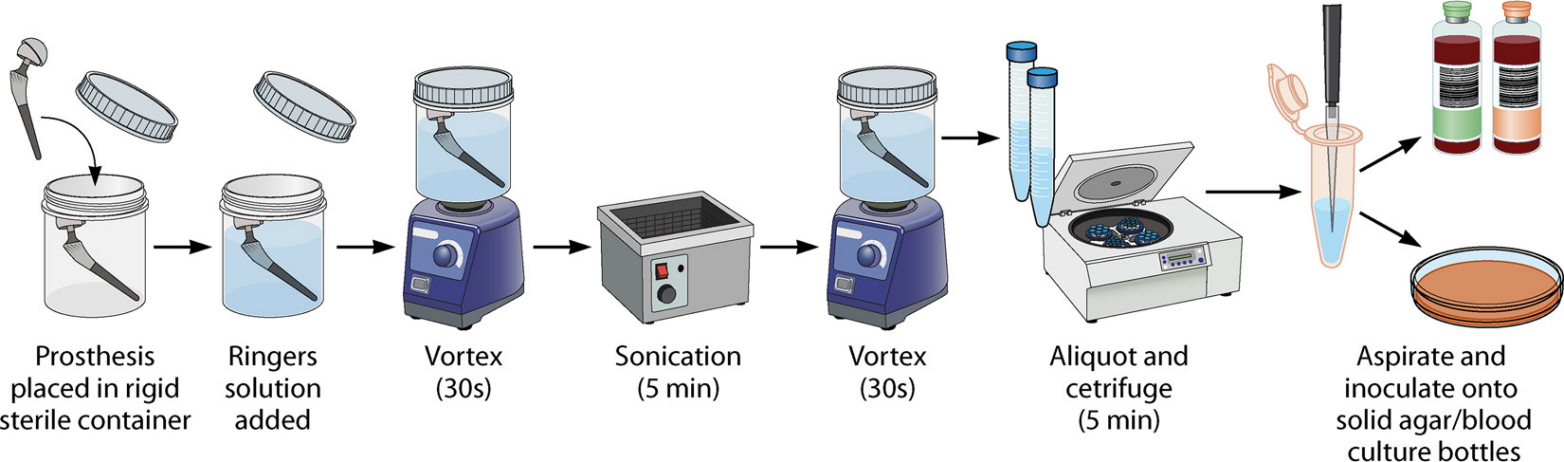
Process of implant sonication.

Tunney et al. described use of this technique in 1998 (18) to disrupt adherent biofilms from explanted prostheses. This technique was subsequently modified with an additional vortexing step as well as utilization of polypropylene containers and evaluated as a diagnostic tool in a prospective trial of 331 patients (19). They demonstrated a sensitivity higher than that of tissue culture (79% versus 61%, respectively) with retained specificity (99%). Sonication-vortexing (Fig. 2) as a diagnostic tool has been evaluated in multiple domains of prosthetic joint infection, as well as other orthopedic implant-associated infections. The majority of published literature reports increased sensitivity with use of sonication in PJI. In their prospective study, Dudareva and colleagues (20) found tissue culture to have a sensitivity higher than that of sonication fluid (69% versus 56%, respectively), although combined sensitivity of both techniques was higher than that of either alone. Notably, this study defined a positive sonicate fluid culture as ≥50 CFU per milliliter (CFU/mL), and with a reduced threshold of 10 CFU/mL, the sensitivities of tissue and sonication fluid culture were 77% (71 to 82) and 72% (66 to 77), respectively (*P *= 0.063). The threshold for positivity used affects both the sensitivity and specificity of sonication fluid culture. A 2014 meta-analysis of 12 studies (21) found that compared with 1, 20, or 50 CFU, a threshold of 5 CFU for diagnosing PJI had the highest sensitivity at 82% (76 to 87) and the highest specificity at 99% (98 to 100).

Identification of organisms detected by culture-based techniques has been transformed by the advent of MALDI-TOF (matrix-assisted laser desorption/ionization time-of-flight) mass spectrometry (MS). Utilizing proteomics for identification of microorganisms allows for rapid identification and has replaced more time- and cost-intensive traditional biochemical assays. Prompt pathogen identification is an important tool of antimicrobial stewardship to ensure antimicrobials are appropriate and minimize duration of empirical rather than pathogen-directed therapy. A recent study by Kuo et al. (22) looked at use of MALDI-TOF MS directly on synovial fluid inoculated in blood culture bottles, an approach which circumvents the need for subculture of specimens prior to identification. Comparing direct and routine MALDI-TOF MS on synovial fluid samples in this study, the direct approach had a faster turnaround time but a lower pathogen identification rate than a standard approach. Direct MALDI-TOF MS identified 85.3% of Gram-positive organisms and 92.3% of Gram-negative organisms compared with standard approach. The lower performance with regard to detection of Gram-positive organisms is particularly significant in relation to orthopedic infection, where the most common causative organisms are Gram positive.

## MOLECULAR TECHNIQUES

**PCR.** PCR has been utilized in multiple domains of infection diagnostics. Use of PCR in the diagnosis of bone and joint infections generally adopts one of two approaches: (i) PCR using primers to target a single organism or a multiplex panel of common causative organisms or (ii) broad-range 16S PCR followed by either sanger or next-generation sequencing (NGS) to identify the causative agent of positive results. The broad-range approach exploits primers targeting highly conserved regions of the bacterial 16S ribosomal subunits. These subunits also contain variable regions which differ between species, allowing identification of bacteria (23). PCR as a diagnostic technique has multiple potential advantages. The speed of bacterial identification can allow prompt initiation of appropriate pathogen-directed antimicrobial agents. Given that the technique can detect both viable and nonviable bacteria, the sensitivity should be less affected by antibiotic administration prior to sampling. It may also detect difficult-to-culture or fastidious organisms.

One disadvantage of the multiplex versus broad-range technique is that the panel will detect only what has been predefined by primer inclusion and, thus, will miss more unusual causes of bone and joint infections. The utility of broad-range PCR with sanger sequencing is lower for polymicrobial infections than that with standard culture or NGS techniques, as presence of multiple organisms results in overlapping reads which are difficult to interpret (24). The role of pathogen-specific PCR depends on the clinical context. Kingella kingae is an important pathogen in pediatric osteoarticular infection, and K. kingae-specific PCR has been demonstrated to have a higher yield in detecting this organism higher than 16S PCR (25). Different PCR techniques have been studied in synovial, tissue, and periprosthetic/sonicate samples with various results. A study by Cazanave et al. (26) designed a PCR panel targeting PJI pathogens and applied it to sonicate fluid samples from hip or knee at revision or resection arthroplasties. Defining positive tissue cultures as those with growth in two or more samples, sonicate fluid PCR was more sensitive than tissue culture and, in the patient cohort who received antibiotics within 14 days of surgery, was also more sensitive than sonicate fluid culture. However, further study of this multiplex assay on tissue samples (27) demonstrated that the sensitivity of PCR on tissue (16%) was much lower than that of tissue culture (69%), synovial fluid culture (72%), and sonicate fluid culture (77%). There are several commercially available multiplex PCR panels which differ in the number and type of primers included. Performance of these assays varies in studies, but they have been reported to have a sensitivity similar to (28) or even lower than (29) that of standard culture. However, there were cases in both studies where multiplex PCR identified organisms not detected by culture methods.

Broad-range PCR followed by Sanger sequencing has been evaluated in diagnosis of PJI on both periprosthetic tissue (30) and sonicate fluid (31). Bémer et al. (30) found a lower sensitivity with PCR than that with standard culture technique, with notably poor sensitivity for polymicrobial infections with almost a quarter of polymicrobial PJIs yielding negative PCR results. Gomez et al. (31) found PCR and culture of sonicate fluid equivalent. More recent studies have looked at 16S PCR combined with NGS. Tarabichi et al. utilized this approach in their study of samples collected at revision and primary arthroplasty (32). While the sensitivity profile in confirmed infection was favorable with microbes detected by NGS in 25 of 28 infected cases, there were also microbes detected in 9 of 36 patients undergoing revision arthroplasty who did not meet infection criteria and had negative cultures. Flurin et al. (33) assessed this technique retrospectively compared with standard culture of sonicate fluid from total elbow arthroplasty. Their study found the combined approach of 16S PCR and NGS more sensitive than standard culture of sonicate fluid for detection of PJI with a specificity of 98%. The higher specificity seen in this study likely relates to the quantification threshold used to differentiate pathogens from background noise.

Given the significant burden of orthopedic infections and potential pitfalls of treatment without pathogen identification, there is certainly a role for these techniques. However, further research guiding patient selection, sampling, and interpretation of results is needed to optimize utilization of this technique in widespread clinical practice. Considering diagnostic stewardship in the approach to their utilization may reserve their use to situations in which blood cultures or preoperative sampling cultures are negative or when cultures are likely to be affected by antecedent antibiotics.

**Shotgun metagenomics.** Shotgun metagenomic next-generation sequencing (mNGS) is a technique in which all nucleic acid in the sample can be detected and then sequenced to identify all organisms present. This approach allows for agnostic pathogen detection and has the potential to significantly enhance diagnostics. The benefits are not limited to this broad capacity for detection of microbes; it can also provide additional information for strain identification, surveillance data, and prediction of antimicrobial resistance (34). Metagenomic NGS strategies may utilize either a DNA- or RNA-based approach or, indeed, both. A DNA-based approach will detect the presence of all organisms other than RNA viruses which require an RNA-based approach. Incorporating an RNA-based approach also allows determination of which organisms are transcriptionally active (35).

Several studies have looked at utilization of this approach in relation to orthopedic infection analyzing either synovial fluid or sonication fluid. Street et al. (36) compared metagenomic sequencing with standard culture in 97 sonicate fluid samples from prosthetic joints or other orthopedic devices and reported a species level sensitivity of 88% (61/69) and specificity of 88% (85/97). Thoendel et al. (37) identified known pathogens in 94.8% (109/115) of culture-positive PJIs, and new potential pathogens were detected in 43.9% (43/98) of culture-negative PJIs. These findings highlight the potential benefit of this tool in “culture-negative” infections, where traditional culture methods fall short due to either the fastidious nature of the organism or prior use of antibiotics.

In relation to PJI, preoperative synovial fluid analysis is an important evaluation tool for defining a management strategy. A meta-analysis (38) evaluated preoperative aspiration culture for diagnosing PJI and found a pooled sensitivity of 72% (95% confidence interval, 65% to 78%). Ivy et al. (39) evaluated metagenomic shotgun sequencing as a technique to detect and identify pathogens in synovial fluid. Techniques to enhance preoperative diagnostic yield could significantly affect patient care. A total of 168 synovial samples were analyzed from total knee arthroplasties which had been classified as culture-positive PJI, culture-negative PJI, or aseptic failure. Of the 25 synovial fluid culture-negative PJIs, mNGS detected pathogens in 4 cases, but in 2 of these cases the organisms were subsequently found with culture of specimens other than synovial fluid. It has been hypothesized that occult infection may play a role in aseptic failures. Of 61 aseptic failure samples, there was 91% correlation with negative culture result, but in four cases additional organisms were detected: Staphylococcus aureus, two *Acinetobacter* spp., and Dolosigranulum pigrum. While all have previously been implicated in PJI, *Acinetobacter* spp. and D. pigrum are also known potential contaminants. Sequencing failed to detect the pathogen in 14 of 82 culture-positive PJI classified samples. In five of these missed identifications, the known pathogen was present in the metagenomic analysis but failed to meet the defined threshold. This highlights a major challenge of this technique, as lowering thresholds for these reads may result in increased sensitivity but at the cost of lower specificity. The overall sensitivity of mNGS in this study was lower than that of standard culture methods. It did yield additional information in the culture-negative PJI and aseptic failures but only in small numbers. This is an important consideration given that the method had a reported cost in the study of several hundred dollars per specimen as well as complex associated methodology.

Metagenomic next-generation sequencing has also been evaluated as a diagnostic tool for bone and joint infection in the pediatric population, an area of significant importance for enhancing diagnostics, given the relative frequency of bone and joint infections and the challenges with invasive sampling. In their single site study of 42 operative culture samples, Ramchandar et al. (40) found that the overall performance of mNGS was similar to that of usual care testing, with mNGS identifying a pathogen in 26 cases (61.9%) and usual care identifying a pathogen in 24 cases (57.1%). There were 4 cases in which mNGS identified a pathogen (2 cases of Neisseria gonorrhoeae arthritis, 1 case of Brevundimonas vesicularis osteomyelitis, and 1 case of Kingella kingae osteomyelitis) where usual care testing (culture and PCR) was negative. There were two cases in which standard diagnostics identified a pathogen (one case of Borrelia burgdorferi detected by PCR and one case of S. aureus detected by tissue culture) where mNGS did not. However, the authors cite that failed detection of S. aureus may have been related to sampling error. While the mNGS did identify a pathogen in a small number of cases that would not otherwise have been detected, this did not translate to a change in clinical management as the empirical antibiotic regimen provided appropriate coverage.

While several studies have highlighted how this tool could be utilized to augment our diagnostic approach, there are some drawbacks to overcome prior to widespread utilization. With a shotgun sequencing approach, the vast majority of nucleic acid in the sample will be human host derived, making pathogen detection difficult (34). Refinement of processes for microbial enrichment and DNA isolation is crucial to its diagnostic use. The technique is also susceptible to bacterial contamination at multiple steps during its processing. Interpretation of such results is difficult, as many contaminating organisms are also potential pathogens in the context of PJIs. Additionally, interpretation of mNGS reads requires bioinformatic pipelines which are also limited by completeness of reference databases. Widespread use of this technique is currently limited by its cost and the complex lab and bioinformatic workflows it requires. In terms of diagnostic stewardship, optimal patient selection, methodology, and interpretation of results, mNGS remains to be fully elucidated to allow the technique to be used in a cost-effective manner which positively affects patient care.

## DETECTION OF HOST RESPONSE

**Serum biomarkers.** Serum inflammatory markers such as erythrocyte sedimentation rate (ESR) and C-reactive protein (CRP) are relatively inexpensive and readily available. However, they lack the specificity of an ideal diagnostic test, as they may be elevated in many systemic illnesses, inflammatory conditions, malignancy, or postsurgical intervention. A normal value does not entirely preclude infection (41), particularly when considering PJI, where the pathogen may be a more indolent organism. This retrospective study from Mayo Clinic reviewed 538 total knee arthroplasties and 414 total hip arthroplasties undergoing surgical intervention for PJI. The preoperative ESR and CRP were normal in only 4% of cases. They report a sensitivity of 81% and 93% for ESR and CRP, respectively, in this cohort.

Other biomarkers of interest in orthopedic infection include interleukin 6 (IL-6) and procalcitonin. IL-6 is produced by activated monocytes and macrophages and stimulates production of several other acute phase reactants. A systematic review of inflammatory markers in PJI (42) found IL-6 to have the highest diagnostic accuracy compared with white cell count, ESR, and CRP, with a reported pooled sensitivity and specificity for IL-6 of 97% and 91%, respectively. A small study evaluating procalcitonin in PJI found that it was not useful in distinguishing infection from aseptic loosening (43). However, a prospective evaluation of procalcitonin in the diagnosis of native joint septic arthritis (44) found that using a cutoff of 0.25 ng/mL resulted in a higher sensitivity and specificity than CRP in diagnosis of septic arthritis. In current clinical practice, available serum biomarkers serve as adjunctive tests which may support or lower clinical suspicion of infection rather than definitive diagnostic tools.

**Synovial fluid analysis.** Synovial fluid sampling is obtained as standard of care in both suspected native and prosthetic joint septic arthritis. Synovial fluid white cell count of 50,000/μL is typically used as the cutoff for native joint septic arthritis (45). However, a recent large study using receiver operating curve analysis suggested a lower threshold of 33,000 cells/μL and 16,000 cells/μL for patients who did or did not receive prior antibiotics, respectively (46). The thresholds used for PJI are lower, and interpretation needs to consider the joint involved, timing of implantation of the joint, and duration of symptoms. A prospective study of patients undergoing revision total knee arthroplasty identified an optimal cut off rate of 1,700 leukocytes/μL, giving a sensitivity of 94% and a specificity of 88% for diagnosing PJI (47). When applied to this study, increasing the cutoff to 2,500 leukocytes/μL reduced sensitivity to 44%. In their retrospective review of synovial fluid aspirates undertaken post total hip arthroplasty, Choi et al. (48) found that synovial fluid white blood cell (WBC) count in arthroplasty patients with symptoms for up to 2 weeks was significantly higher than that in patients with symptoms for more than 2 weeks. A 2018 meta-analysis (49) evaluated accuracy and yield of synovial fluid analysis in PJI including both hips and knees. Only 10 articles met inclusion criteria but reported an optimal threshold of 3,000 leukocytes/μL, concluding that lower cutoffs were associated with lower specificity. However, they report that due to inadequate number of clinical studies involving total knee replacements, precise assessment of accuracy of synovial fluid analysis in detecting total knee arthroplasty infection was precluded. Interpretation of synovial fluid analysis in both native and prosthetic joint arthritis is complicated in the setting of inflammatory arthropathies. Thus, interpretation of synovial fluid analysis should consider the clinical context, patient comorbidities, and immune status, and for prosthetic joints, the duration of symptoms, timing relative to surgery, and joint involved.

Several biomarkers have been evaluated in synovial fluid assessing for the host inflammatory response at the site. Of the known biomarkers, leukocyte esterase and alpha defensin have been studied extensively. Leukocyte esterase is an enzyme present in neutrophils and is readily available as a colorimetric testing strip. A prospective evaluation (50) of leukocyte esterase testing on synovial fluid aspirates from patients undergoing revision hip or knee arthroplasty for either mechanical failure or infection assessed sensitivity and specificity using Musculoskeletal Infection Society (MSIS) criteria as the diagnostic standard. Utilizing ++ as a positive result, reported sensitivity and specificity were 66% and 97.1%, respectively. This study acknowledges that this relatively low sensitivity does not support its use as an independent screening method. The utility of leukocyte esterase testing strips is significantly affected by presence of blood in sample (51), which is problematic, as many synovial fluid samples will be contaminated with blood.

Defensins are peptides produced in response to microbes or proinflammatory cytokines, and alpha defensin has been studied as a biomarker of PJI. The test is available as a lateral flow test and enzyme linked immunosorbent assay (ELISA). The lateral flow test has the advantage of use as a rapid point of care test with results available in 10 minutes. A meta-analysis (52) of synovial biomarkers in 2018 reported a pooled sensitivity of 97% for alpha defensin ELISA with a specificity of 97% and a sensitivity of 80% with specificity of 89% for the lateral flow test kits. While only four studies of alpha defensin ELISA were included, a sensitivity higher than that of other biomarkers (synovial CRP, IL-6, and leukocyte esterase) was reported, supporting its use as an adjunctive diagnostic test. A recent study evaluated MALDI-TOF MS as a technique to measure alpha defensin in synovial fluid. Iorio et al. (53) evaluated samples from 138 patients undergoing revision for either infective or aseptic cause and found that detection of alpha defensin using MALDI-TOF MS had a sensitivity and specificity of 93% and 98%, respectively, using 2018 MSIS criteria as the reference standard. They did not compare the accuracy of this method with that of either lateral flow or ELISA in the study samples, and further study with larger case numbers is required to determine the reliability and reproducibility of MALDI-TOF MS in measurement of alpha defensin in synovial fluid.

**Histology.** Histological analysis of bone and synovial specimens confirms presence of inflammatory infiltrate and can provide important information regarding underlying cause. Histological analysis is also useful in PJI, particularly, as it is not as affected by preoperative antibiotics. Various criteria have been used to define PJI from a histopathologic perspective utilizing different thresholds of neutrophils per high-power field. Morawietz et al. evaluated optimal thresholds to distinguish infection from aseptic loosening on operative samples and concluded using receiver-operator curves that the optimal threshold was ≥23 neutrophils per 10 high-power fields, giving a sensitivity of 77% and specificity of 97% compared with clinical diagnosis (54). Criteria based on examination of a larger number of high-power fields may avoid overcalling focal areas of inflammation which may be seen with mechanical stress (54). These criteria were further evaluated in a multicenter trial (55) comparing this criterion with previously published criteria by the same group which subtyped changes in the periprosthetic membrane into four different categories (56) and found that it had a slightly higher sensitivity and specificity. Histological analysis of intraoperative frozen sections can be used to guide decision-making regarding surgical approach; however, the sensitivity of histopathology alone is not high enough to rule out prosthetic joint infection if there are other factors suggesting infection.

**Radiological imaging.** Radiological imaging aids in both diagnosis of orthopedic infection and assessment of complications. Particularly with implant-related infection where clinical signs may be subtle, suggestive radiographic findings can be helpful in providing evidence to support and guide further invasive diagnostic sampling.

**FIG 3:**
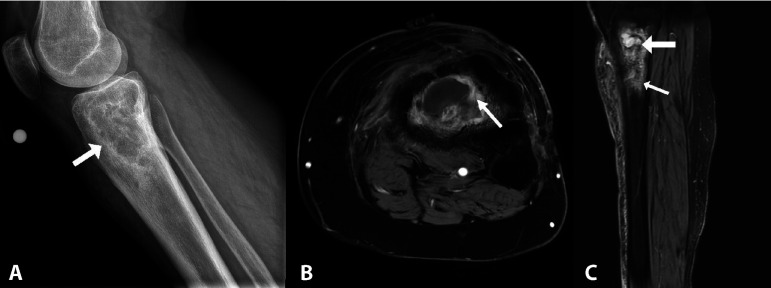
Plain film (A) demonstrating chronic tibial osteomyelitis with intraosseus abscess. MRI images from the same patient demonstrating intense peripheral enhancement consistent with a granulation layer (B, axial sequence post gadolinium contrast administration) and internal fluid signal (larger arrow) and marked peripheral edema (smaller arrow) again suggestive of intraosseus abscess (C, sagittal T2 fat-saturated sequence).

In general, the yield of plain films is relatively low, but they are inexpensive, are widely available, and may be useful for anatomic outlines and providing supportive evidence of infection. Radiographic changes related to osteomyelitis (Fig. 3) can take several weeks to develop but once established may demonstrate focal osteopenia, periostitis, and ultimately development of a sequestrum within the bone. In relation to PJI, plain films are also useful to evaluate for other causes of pain related to the implant, such as periprosthetic fracture, dislocation, or implant loosening. Ultrasonography also has the advantage of widespread availability as well as avoiding ionizing radiation. It is generally not helpful in the workup of osteomyelitis but is useful in assessing joint effusions and can guide synovial fluid aspiration, providing additional valuable diagnostic information.

Computed tomography (CT) provides enhanced spatial evaluation of both the soft tissue and bone compared to conventional radiography but is less sensitive than magnetic resonance imaging (MRI) in the evaluation of osteomyelitis. It may demonstrate soft tissue changes or fluid collections suggestive of infection. Both CT and MRI are impeded by artefactual changes from prostheses in the setting of implant-associated orthopedic infections. MRI is the imaging modality of choice in assessment of osteomyelitis; as well as enhanced sensitivity over CT, it can detect changes related to bone marrow edema earlier in the course of infection. MRI changes can persist for weeks and months posttreatment.

Nuclear imaging may be utilized as a diagnostic adjunct in the evaluation of PJI, as it can assess for changes associated with inflammation while avoiding artifact-related issues seen with other modalities. Bone scintigraphy is one of the most widely utilized of these modalities in assessment of prosthetic joints but reported accuracy ranges between 50 and 70% (57). This imaging technique uses three phases, and abnormalities detected correlate with the rate of bone turnover. This results in limited utility in diagnosis of PJI in the early postoperative period. Combined white blood cell and marrow imaging may also be used to differentiate aseptic loosening from infection when other modalities are unavailable or nondiagnostic. Published data on use of [^18^F]fluoro-2-deoxyglucose positron emission tomography in diagnosis of PJI are variable (57), but if cost is not a barrier, in some cases it may be more accessible and more convenient for patients than other nuclear medicine techniques.

## CONCLUSION

Diagnostic approaches in orthopedic infection are multifaceted, and enhancing diagnostic yield requires a collaborative approach involving orthopedic surgeons, radiologists, microbiologists, and infectious diseases specialists. Strengths and limitations of various diagnostic tests are summarized in Table 1. Despite significant advances, there is no one single perfect diagnostic test. Culture-based techniques remain at the core of diagnostics, but these must be preceded by a fastidious sampling approach and can be augmented by molecular techniques in select cases. Future research should focus on further development of novel molecular diagnostics. However, the use of existing culture-based diagnostics must continue to be optimized and a stewardship mindset must be applied to any new molecular diagnostic test. Careful assessment of the inflammatory response must include radiologic imaging, blood, synovial fluid, and histologic testing to define the presence of an infectious syndrome. Just as with molecular diagnostics, any new tool for evaluation of the host inflammatory response should be carefully evaluated in comparison to already available techniques. Ultimately, the future diagnosis of orthopedic infections will continue to rely upon a global assessment of all available microbiological and nonmicrobiological data.

**TABLE 1:** Summary of strengths and limitations of strategies used in detection of pathogen and host response

Detection strategy	Strengths	Limitations
Detection of pathogen		
Culture-based techniques	Mainstay of pathogen detection in orthopedic infection Increased yield with inoculation of synovial fluid, periprosthetic tissue, and sonicate fluid samples into blood culture bottles Allows for antimicrobial susceptibility profiling of identified organisms Widely available	In setting of PJI, multiple samples required due to low sensitivity of single sample as well as difficulty distinguishing contaminants from true pathogens Yield diminished by presampling antibiotic administration Prolonged incubation required for detection of fastidious organisms
PCR	Facilitates rapid pathogen detection Useful in culture-negative cases where there may have been presampling antibiotic administration Can be used to detect antimicrobial resistance genes	Use of multiplex diagnostic panels will miss atypical pathogens
Shotgun metagenomics	Agnostic pathogen detection Can provide additional information for strain identification, surveillance data, and prediction of antimicrobial resistance	Significant cost Complex associated workflow Technique susceptible to bacterial contamination at multiple steps during processing
Detection of host response		
Serum biomarkers	Inexpensive Widely available	Lack specificity in diagnosis of orthopedic infection
Synovial fluid cell count and differential	Quantitative assessment of joint inflammation useful in both native and prosthetic joint infection	Difficult to interpret in setting of inflammatory arthropathies Varies depending on presence of prosthesis, duration of symptoms/time postimplantation/joint involved
Synovial fluid biomarkers	Alpha defensin available as both a lateral flow test (result available within minutes) and an ELISA Alpha defensin higher reported sensitivity compared with CRP, IL-6, and leukocyte esterase	Utility of leukocyte esterase test affected by presence red cells Costly Lateral flow alpha defensin lower sensitivity compared to ELISA
Histology	Can confirm presence inflammation and give further information regarding potential etiology Intraoperative frozen section can aid real-time decision-making regarding surgical approach in setting of revision of prosthetic implants	Sensitivity not high enough to use as a stand-alone “rule out test” for infection
Radiology	Useful in evaluation of noninfective causes of symptoms Can provide supportive evidence for infection May guide invasive diagnostic sampling	Findings often nonspecific
